# The Effect of Patient Narratives on Information Search in a Web-Based Breast Cancer Decision Aid: An Eye-Tracking Study

**DOI:** 10.2196/jmir.2784

**Published:** 2013-12-17

**Authors:** Victoria A Shaffer, Justin Owens, Brian J Zikmund-Fisher

**Affiliations:** ^1^University of MissouriDepartment of Health Sciences, School of Health ProfessionsDepartment of Psychological Sciences, College of Arts & ScienceColumbia, MOUnited States; ^2^Wichita State UniversityDepartment of PsychologyWichita, KSUnited States; ^3^University of MichiganDepartment of Health Behavior & Health EducationDepartment of Internal Medicine (General Medicine)Ann Arbor, MIUnited States

**Keywords:** personal narratives, decision aids, eye tracking, breast cancer

## Abstract

**Background:**

Previous research has examined the impact of patient narratives on treatment choices, but to our knowledge, no study has examined the effect of narratives on information search. Further, no research has considered the relative impact of their format (text vs video) on health care decisions in a single study.

**Objective:**

Our goal was to examine the impact of video and text-based narratives on information search in a Web-based patient decision aid for early stage breast cancer.

**Methods:**

Fifty-six women were asked to imagine that they had been diagnosed with early stage breast cancer and needed to choose between two surgical treatments (lumpectomy with radiation or mastectomy). Participants were randomly assigned to view one of four versions of a Web decision aid. Two versions of the decision aid included videos of interviews with patients and physicians or videos of interviews with physicians only. To distinguish between the effect of narratives and the effect of videos, we created two text versions of the Web decision aid by replacing the patient and physician interviews with text transcripts of the videos. Participants could freely browse the Web decision aid until they developed a treatment preference. We recorded participants’ eye movements using the Tobii 1750 eye-tracking system equipped with Tobii Studio software. A priori, we defined 24 areas of interest (AOIs) in the Web decision aid. These AOIs were either separate pages of the Web decision aid or sections within a single page covering different content.

**Results:**

We used multilevel modeling to examine the effect of narrative presence, narrative format, and their interaction on information search. There was a significant main effect of condition, *P*=.02; participants viewing decision aids with patient narratives spent more time searching for information than participants viewing the decision aids without narratives. The main effect of format was not significant, *P*=.10. However, there was a significant condition by format interaction on fixation duration, *P*<.001. When comparing the two video decision aids, participants viewing the narrative version spent more time searching for information than participants viewing the control version of the decision aid. In contrast, participants viewing the narrative version of the text decision aid spent less time searching for information than participants viewing the control version of the text decision aid. Further, narratives appear to have a global effect on information search; these effects were not limited to specific sections of the decision aid that contained topics discussed in the patient stories.

**Conclusions:**

The observed increase in fixation duration with video patient testimonials is consistent with the idea that the vividness of the video content could cause greater elaboration of the message, thereby encouraging greater information search. Conversely, because reading requires more effortful processing than watching, reading patient narratives may have decreased participant motivation to engage in more reading in the remaining sections of the Web decision aid. These findings suggest that the format of patient stories may be equally as important as their content in determining their effect on decision making. More research is needed to understand why differences in format result in fundamental differences in information search.

## Introduction

Narratives (ie, personal stories about health experiences) are a popular vehicle for patients to learn about diseases and treatments, and they are commonly available on the Internet, on social media sites, and in patient decision support tools [1,2]. The proliferation of patient stories through Web-based outlets has provided researchers cause for concern given that there are strong theoretical reasons to believe that narratives have powerful effects on decision making [3,4]. Research on attitudes and persuasion suggests that patient stories are likely to be more influential than other message formats because (1) narratives have the ability to “transport” the reader or viewer into the story [5,6], (2) narratives are likely to cause greater elaboration [7], and (3) people are more likely to rely on case studies than statistical information [8]. Further, research on dual process models and the affect heuristic suggest that narratives may facilitate different modes of information processing than other message formats, which could result in greater weight given to narrative information during the decision process [9-14].

Although several recent studies have explored the role of narratives, there is still little consensus about how patient stories impact decisions about health care [3,15-22]. One likely reason for the discrepant findings is that many of these studies focused on outcomes such as treatment intentions or decisions. However, recent work suggests that narratives may also influence decision processes such as information search. Therefore, uncovering the true impact of narratives involves measuring decision processes along with outcome measures [17].

In this paper, we report the results of a study examining the effect of patient narratives on information search in a Web-based decision aid during a hypothetical breast cancer treatment task. Women in this study were asked to imagine they had been diagnosed with early stage breast cancer and needed to choose between two surgical treatments—lumpectomy with radiation or mastectomy. Participants were provided access to a Web-based decision aid about surgical treatments for early stage breast cancer and were allowed to freely browse the aid until they developed a preferred surgical treatment. Some of the women viewed a decision aid with patient narratives; others viewed a decision aid without narratives. To track information search, we monitored participants’ eye movements while viewing the Web decision aid.

Our primary research question was whether including narratives in Web decision aid would alter information search strategies. Broadly, we hypothesized that providing patient stories would increase the breadth and depth of information search. Without narratives, we expected that participants would focus on information related to survival (eg, local recurrence and survival rates). By contrast, we predicted that patient stories about their decision process and their experiences with treatment would draw attention to other attributes of the treatments that are unrelated to survival but may be important for subjective experience and post-treatment satisfaction (eg, length of recovery time). Therefore, we predicted participants would spend more time searching for information discussed in the narratives.

## Methods

### Recruitment

We recruited 56 women from Wichita, Kansas, via ads in a local newspaper and on the Wichita State University website. Participants were required to be 18 years of age or older, be native English speakers, and have normal or corrected-to-normal vision. Participants were excluded from the study if they were pregnant, had previously been diagnosed with breast cancer, reported wearing bifocal or trifocal corrective lenses, reported wearing lenses with reflective coatings, or had serious eye injuries. The age and pregnancy restrictions were chosen to avoid recruiting members of vulnerable populations, as defined by the Institutional Review Board. Native English speakers were recruited to ensure adequate comprehension of the material presented in the Web decision aid. We restricted the sample to women without a personal history of breast cancer because we wanted to avoid biases in information search due to prior treatment choices. We also avoided recruiting participants with major vision problems because of the difficulties associated with eye tracking calibration.

Screening for these criteria was conducted via an online survey. Invitations to participate in the study were extended to a portion of those who completed the survey and met the eligibility requirements. However, preference was given to older adults to more accurately reflect the distribution of age among new breast cancer patients. Participants were compensated US $100 for their study participation.

### Study Materials

Participants in this study were asked to imagine that they had been diagnosed with early-stage breast cancer following the detection of a lump during a breast self-exam. A 1-page description of the diagnostic process was provided; see Multimedia Appendix 1. To help with the treatment decision, participants were given one of four versions of the Web decision aid; assignment to condition was random. They were instructed to take as much time viewing the website as necessary. When they finished viewing the Web decision aid, participants indicated their preferred treatment using a 7-point Likert scale (1=Extremely likely to choose lumpectomy with radiation, 7=Extremely likely to choose mastectomy).

### Web Decision Aid

The Web decision aid used in the study was constructed from a US website produced by Health Dialog that provides information about surgical treatments for early stage breast cancer and includes video interviews with patients and physicians. This website is the companion to their video decision aid, BCR001 v03. All versions of the Web decision aid included 17 pages of content that provided detailed information about lumpectomy and mastectomy using text, images, and (in some versions) videos. See Multimedia Appendix 2 for a screenshot of the first page of the Web decision aid.

To examine the effect of patient narratives, we created two versions of the decision aid. In the two video conditions, participants were provided access to video controls that allowed them to play, pause, and move to different positions in the video timeline. The *video narrative* version of the Web decision aid was a replica of the Health Dialog website, which included videos of patients sharing their stories about deciding between treatments and their experiences with those treatments. The decision aid also included videos of physicians providing didactic information about early stage breast cancer and the treatments. There were 18 videos of interviews with patients or physicians included in this version of the decision aid. Videos were embedded in 7 of the 17 webpages in the decision aid. These pages provided information about (1) decisions to be made after being diagnosed with breast cancer, (2) different types of breast cancers, (3) pathology reports, (4) mastectomy, (5) lumpectomy and radiation, (6) lymph node surgery, and (7) working with your doctor. Many of these videos were clips of interviews from a single patient or physician. However, several videos included clips from multiple patient and/or physician interviews. The narratives were not tailored to the participants with respect to race, ethnicity, or other individual characteristics.

To create the *video control* version of the decision aid, we removed any videos containing patient interviews. However, the four video clips of physician interviews remained in the decision aid. These videos appeared on pages that provided information about (1) decisions to be made after being diagnosed with breast cancer, (2) different types of breast cancers, (3) pathology reports, and (4) lymph node surgery.

To distinguish between the effect of narratives and the effect of videos, we created two additional versions of the Web decision aid. We constructed a *text narrative* version of the Web decision aid by replacing the patient and physician interviews with text transcripts of the videos and a *text control* version by removing the transcripts of the patient stories. Examples of two video transcripts are provided in Multimedia Appendix 3.

Participants were randomly assigned to view one of the four Web decision aids (video narrative, video control, text narrative, or text control); however, we overweighed assignment to the video conditions, with about two-thirds of participants viewing either the video narrative or video control decision aids (n=36). We chose to oversample the video conditions because they represented the naturalistic version of this decision aid. About one-third of participants (n=20) were randomized to one of the two text decision aids (text narrative or text control).

### Eye Tracker

To assess information search in the Web decision aid, we used a Tobii 1750 eye-tracker integrated with a 96dpi 17-inch monitor to record participant eye movements. The Tobii 1750 samples eye movements every 20 milliseconds and has a spatial resolution of .25° and a spatial accuracy of .5°. The resolution of the monitor was set to 1280 x 1024 pixels. This eye tracker is situated nonintrusively within the monitor enclosure and provides the ability to record eye movements without the use of a chinrest or head mount. In conjunction with the eye-tracker, an Intel-based computer with 2GB of RAM running Microsoft Windows Vista was used to operate the eye-tracker. All participants were calibrated on the eye-tracker using the Tobii Studio 9-point calibration routine to aid tracking accuracy. Eye movements during the study were recorded with Tobii Studio software.

### Statistical Analyses

To create manageable units of analysis, we a priori divided the Web decision aid into 24 areas of interest (AOIs). Most webpages within the decision aid were defined as a single AOI. However, some webpages contained multiple subheadings with content directly related to material covered in the patient narratives. Because we wanted to assess whether information search differed for material explicitly discussed in the narratives, we examined these areas separately. To do this, we defined multiple AOIs on webpages where this occurred. For example, the webpage describing mastectomy contains several subheadings including what to expect after mastectomy, appearance after mastectomy, and local recurrence after mastectomy. Because there were two patient stories about appearance after mastectomy on this page, we expected information search about appearance to differ from information search about local recurrence for the conditions with patient stories. Therefore, we defined these three subheadings as separate AOIs within the page.

The primary outcome in this study was total fixation duration per AOI, measured in milliseconds. This represents the total amount of time that a participant was looking at information in a predefined AOI, yielding 24 total fixation duration measurements per person. Our measures of fixation duration exclude looking time associated with the physician and patient interviews. Each of the interviews, whether they were a video or a transcript, opened a separate webpage that was not one of the predefined AOIs. Time spent viewing these pages was not included in the analyses so that we could focus on differences in information search in the remaining sections of the Web decision aids that were common across conditions. Fixation duration was log transformed to reduce the positive skew in the distribution. Our secondary outcome was treatment preference.

We used multilevel modeling to examine the impact of narratives on information search. For these analyses, the log-transformed fixation durations associated with the 24 AOIs were nested within study participants (N=56) providing over 1300 measurement occasions. We tested models with both random and fixed slopes and intercepts with AOI as the Level 1 variable and participant as the Level 2 variable. Condition (patient stories present vs patient stories absent) and format (text vs video) were included as Level 2 predictors. Multilevel models were assessed using the restricted maximum likelihood estimate with the lme4 package in R [23]. The best model, as judged by Akaike information criterion and Bayesian information criterion, is described below. All analyses were two-tailed and a *P* value of .05 was considered significant.

**Figure 1:**
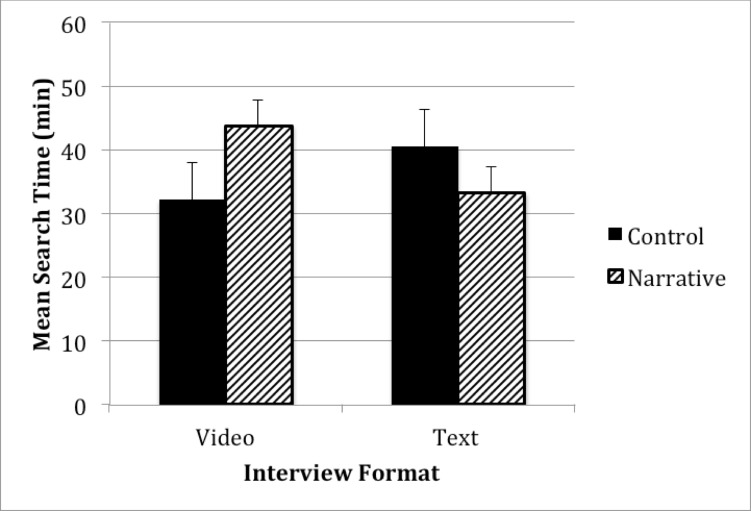
The effect of patient narratives on information search in a Web decision aid (error bars represent 1 SE).

In this model, there was a significant main effect of condition, *P*=.02. Participants viewing decision aids with patient narratives spent more time searching for information (mean 39.88 min, SD 15.62 min) than participants viewing the decision aids without narratives (mean 35.08 min, SD 16.09 min). The main effect of format was not significant, *P*=.10. However, there was a significant condition by format interaction on fixation duration, *P*<.001; see Figure 1 for an illustration of this effect. When comparing the two video decision aids, participants viewing the narrative version spent more time searching for information than participants viewing the control version of the decision aid. The opposite pattern emerges when comparing the two text versions of the decision aid. Participants viewing the narrative version spent less time searching for information than participants viewing the control version of the decision aid.

Despite the difference in search time between conditions, the difference in treatment preference between the control and narrative decision aid conditions was not significant, *t*_54_=0.98, *P*=.33. However, it should be noted that with this sample size, the study was powered to detect only a large effect (Cohen’s *d*=0.8) for this outcome measure.

## Results

### Participants

Participants ranged from 30-71 years of age (mean 48.7, SD 9.4). Of the sample, 96% (54/56) were Caucasian, and 74% (41/56) were college graduates. Although no participants had a personal history of breast cancer (this was an exclusion criterion), approximately 41% (23/56) of participants had a family history of breast cancer and 64% (36/56) had a friend who had previously been diagnosed with breast cancer. See Table 1 for complete participant demographics.

**Table 1:** Participant characteristics (N=56).

Characteristics			n (%)
**Age, years (mean 48.7)**			
	30-39	10 (17.9)	
	40-49	20 (35.7)	
	50-59	20 (35.7)	
	Above 60	6 (10.7)	
**Race**			
	White	54 (96.4)	
	Black or African American	2 (3.6)	
**Education**			
	High school graduate	6 (10.8)	
	Some college	9 (16.1)	
	College graduate	21 (37.5)	
	Some graduate school	2 (3.6)	
	Graduate degree	18 (32.1)	
**Income (in USD)**			
	Less than $30,000	6 (10.7)	
	$30,001 to $50,000	11 (19.6)	
	$50,001 to $75,000	17 (30.4)	
	$75,001 to $100,00	13 (23.2)	
	Above $100,000	9 (16.1)	
**History with breast cancer**			
	Family history of breast cancer	23 (41.1)	
	Friends with breast cancer	36 (64.3)	

### Effect of Narratives on Fixation Duration

The best fitting multilevel model was a random intercept, fixed slope model. The intercept varied by webpage and individual, and slopes were fixed for condition (control vs narrative), format (text vs video), and their interaction. Parameter estimates for the model are provided in Table 2.

**Table 2:** Parameter estimates for the multilevel models of log-transformed fixation duration.

Parameters	β	SE	*t*	*P*
Intercept	10.92	0.20	55.25	<.001
Condition (control vs narrative)	0.36	0.15	2.43	.02
Format (text vs video)	0.29	0.18	1.64	.10
Condition x Format	0.80	0.25	3.23	.001

### Use of Narratives

Although the patient narratives were available to all participants in the narrative conditions, not all participants chose to view the narratives while reviewing the decision aid. Table 3 describes the content of each narrative, indicates the proportion of times that narrative was viewed in both the text and video narrative conditions, and displays the mean time spent on the webpage with that narrative. Note each narrative was located on a separate webpage that would open when users clicked on the content. This allowed us to track use of narrative information separately from information search in the rest of the decision aid. The narratives were accessed at a similar rate in both the text and video narrative conditions. However, participants in the video narrative conditions spent more time with the narratives than participants in the text narrative conditions.

**Table 3:** Time spent viewing the patient and physician interviews.

			Text narratives (n=10)		Video narratives (n=17)	
Title of content	Includes patients	Includes physicians	% View (n)	Mean time on page, seconds	% View (n)	Mean time on page, seconds
Description of Diagnosis	✔	✔	60 (6)	67.28	47 (8)	117.19
A Woman’s Preference is Important		✔	50 (5)	18.79	35 (6)	30.92
Take Your Time Making a Decision	✔	✔	60 (6)	27.96	35 (6)	87.09
Types of Breast Cancer		✔	30 (3)	20.93	59 (10)	44.70
Pathology Report		✔	40 (4)	16.39	59 (10)	36.37
Why Two Patients Chose Mastectomy	✔		50 (5)	23.74	65 (11)	48.98
Appearance After Mastectomy	✔		80 (8)	21.24	53 (9)	50.57
Breast Reconstruction	✔		70 (7)	7.75	53 (9)	19.14
Why Patient A Chose Lumpectomy	✔		50 (5)	5.00	53 (9)	15.11
Why Patient B Chose Lumpectomy	✔		50 (5)	11.38	59 (10)	27.86
Radiation After Lumpectomy	✔	✔	50 (5)	23.50	65 (11)	57.71
Side Effects of Radiation	✔		40 (4)	15.71	47 (8)	30.15
Appearance After Mastectomy	✔		50 (5)	17.60	41 (7)	29.10
Sentinel Node Biopsy		✔	30 (3)	34.32	47 (8)	51.77
Side Effects of Lymph Node Surgery	✔		40 (4)	16.62	41 (7)	35.16
Being Involved in Treatment Decisions	✔		70 (7)	8.13	53 (9)	28.64
Making Decisions	✔		60 (6)	25.29	47 (8)	73.42
Life After Breast Cancer	✔		50 (5)	15.71	53 (9)	47.56

### Local Versus Global Differences in Search Patterns

The inclusion of random slopes did not improve the fit of either model suggesting the pattern of information search described above is consistent across AOIs. Although participants spent more or less time in a particular AOI, the relative difference in search time between groups remained constant. Although we predicted that patient stories would influence search for specific pieces of information (eg, appearance after surgery), the narratives instead appeared to influence information search more globally. These search patterns are illustrated in Multimedia Appendices 4 and 5. Both appendices display heat maps depicting group differences in information search patterns on a summary page of the Web decision aid that compares the two treatments. Heat maps use color to illustrate fixation duration for a specific section of a text or image. The colors of a heat map should be interpreted like radar maps used by meteorologists where color represents precipitation intensity as an increasing ordinal function from blue to purple to white.

Multimedia Appendix 4 illustrates differences in search for information about local recurrence, and Multimedia Appendix 5 shows differences in search for information about appearance after surgery. In both appendices, narratives increase information search in the video conditions but decrease information search in the text conditions. We chose these two AOIs because they differ with respect to the amount of time these topics were discussed within the patient narratives. Specifically, appearance was mentioned more frequently than local recurrence. Thus, the fact that the same information search patterns exist in both AOIs provides evidence for the impact of narratives on global (across the entire decision aid) rather than local (within a specific section of the decision aid) information search.

## Discussion

### Principal Findings

This study was designed to examine the impact of patient narratives on information search in a Web-based decision aid. Using an eye tracker to gather search data, we found that the presence of patient narratives increased search time by more than 4 minutes. Further, this increase in search time was seen globally (across all pages of the Web decision aid) and was not specific to pages covering information addressed in the narratives.

In addition, we assigned a subsample of our participants to two text-based versions of the Web decision aids, one with patient stories and one without. This allowed us to examine the effect of narrative format and the interaction between narrative presence and format on information search. There was no significant main effect of format; however, there was a significant narrative presence by format interaction. While the presence of video-based narratives increased information search, text versions of the patient stories decreased search time. Again, the differences in search patterns between the versions with and without narratives were not limited to areas of the decision aid that included narratives or specifically addressed issues discussed in the narratives.

The difference in the effect of video and text-based patient narratives is somewhat surprising. Although we anticipated that there might be differences in the magnitude of the effects of video and text-based stories, we had not predicted that the direction of the effects would also differ. However, there is a fairly parsimonious, albeit post hoc, explanation for the observed differences. Reading involves a significantly greater amount of cognitive resources than simply watching. Reading requires more active involvement with the material, while watching typically requires only passive involvement. Because of this, we hypothesize that the more effortful act of reading patient narratives decreased participant motivation to engage in more reading in the remaining sections of the Web decision aid. In contrast, watching the patient narrative videos may have had the opposite effect on cognitive load by giving participants a break from reading. This is supported by Table 3, which shows that participants in the video narrative conditions spent more time viewing the narratives than participants in the text narrative conditions. This may have allowed one of the major messages of the stories, “take your time in making a decision”, to have a greater effect on information search by motivating participants to ensure they were fully informed about the treatments.

### Limitations

This study had some limitations that may affect the generalizability of the results. First, participants in the study were engaged in a hypothetical decision task that may differ from the decision process associated with a “real” treatment decision. Second, because we used a decision aid that had been previously produced for other purposes (ie, clinical not experimental use), we were able to manipulate only the presence of patient stories, not their content. Third, our sample had very limited racial and ethnic diversity. Future research is needed to replicate these effects in a more heterogeneous sample.

A recent paper outlining a taxonomy of patient narrative types [17] argues that the precise effect of a patient story is closely tied to its purpose for inclusion in the decision aid, its specific content, and its evaluative valence. Because we did not systematically manipulate these dimensions in this study, we can only speculate about how these characteristics of the stories used in this decision aid may have impacted our results. The content of the stories could be best characterized as a mixture of “process” (ie, descriptions of the process of making a treatment decision) and “experience” (ie, descriptions of a treatment experience) narrative elements. Recent work has shown that process narratives can increase information search in an artificial lab search task [24]. Thus, some of the impact of the video narratives may be due to the process elements covered in the stories. Future studies should examine the specific impact of these three dimensions on information search.

Finally, only about one-third of our sample was used to explore the impact of text-based patient narratives on information search. Although this still resulted in a very rich dataset due to the number of observations nested within each participant, future work should focus on replicating this finding and testing our post hoc hypotheses about the relationship between the cognitive effort associated with reading versus watching narratives and information search.

### Conclusions

In a Web decision aid, videos of patient stories embedded within the larger text-based decision aid increased information search during a hypothetical breast cancer decision task. However, transcripts of the patient videos had the opposite effect. Participants spent about 5 fewer minutes searching for information in the Web decision aid when text-based narratives were included. These findings suggest that the format of patient stories may be equally as important as their content in determining their effect on decision making. Although it is unsurprising that video narratives would be more engaging than text-based narratives (as evidenced by the amount of time spent with each in this study), it is instructive that this engagement generalizes to the remainder of the Web decision aid, which itself was text-based. This suggests that engagement may not be specific to modality, and video narratives may be used to increase involvement with both text and video formats of health messaging. Although video narratives are more expensive to create than text versions, the additional funds may be cost-effective when engagement with the material is critical. However, more research is needed to replicate these findings and to test our hypotheses about why differences in format result in fundamental differences in information search.

## Multimedia Appendix 1

Scenario used in the study describing the diagnostic process.

## Multimedia Appendix 2

Example webpage from the decision aid.

## Multimedia Appendix 3

Transcripts of patient and physician interviews from decision aid (patient and physician names removed).

## Multimedia Appendix 4

Heat maps of an information search on a webpage comparing the two treatments (this section of the webpage highlights differences in local recurrence between the two treatments).

## Multimedia Appendix 5

Heat maps of an information search on a webpage comparing the two treatments (this section of the webpage highlights differences in appearance post-surgery between the two treatments).

## Abbreviations

AOI: area of interest
