# Supplementary material for: The Effect of Patient Narratives on Information Search in a Web-Based Breast Cancer Decision Aid: An Eye-Tracking Study
Source: J Med Internet Res. 2013 Dec 17;15(12):e273. doi: 10.2196/jmir.2784 (PMC3875892; doi:10.2196/jmir.2784)
Supplement: Supplementary file 1 [file jmir_v15i12e273_app1.pdf]

Multimedia Appendix 1. Scenario used in the study describing the diagnostic process.

FOR THE PURPOSES OF THIS STUDY, PLEASE IMAGINE YOURSELF IN THE FOLLOWING SCENARIO:

You have recently discovered a lump in your breast during a routine breast self-exam. Following this discovery, you made an appointment with your primary care physician to have the lump examined. After examining the lump, your physician arranged for you to have a mammogram, which is an x-ray of your breast. The radiologist reading the mammogram characterized the lump as suspicious and recommended a biopsy of the lump. Your physician then referred you to the local Breast Center, a facility specializing in the diagnosis and treatment of breast cancer, to have the lump biopsied. A breast biopsy involves removing a small section of breast tissue for examination. After the biopsy, the tissue sample was sent to a pathologist, a person who diagnoses disease by examining organs, tissues, bodily fluids, etc. The pathologist's job is to determine whether the cells were malignant (i.e. cancerous) or benign (i.e. not cancerous).

You returned to the Breast Center to discuss the results of the biopsy. You were informed that the lump was malignant, and you were referred to an oncologist at the Breast Center for treatment. An oncologist is a physician who organizes the care of cancer patients. After reviewing the reports from the biopsy and mammogram, your oncologist informed you that you have early stage breast cancer. Typically, early stage breast cancer is treated through local therapy—one of two types of breast surgery. Local therapy is often followed by systemic therapy, such as chemotherapy, which is a course of treatment that travels through the blood stream, affecting cells all over the body.

There may be several treatment decisions to make; however, the oncologist explained that the first decision you must make is what type of surgery to have. The other decisions will be made after viewing the pathology report from your surgery. Based on estimates of the tumor size from the biopsy and mammogram, the oncologist informed you that you have two surgical options. In some medical situations, there is a clear right answer, and your doctor can tell you what is best to do. In other situations, like with early stage breast cancer, there are different choices that are reasonable. What is "best" depends upon how you feel about the good and bad things that might happen with each choice. In this case, you do have a choice.

There are two surgeries designed to remove cancer from your breast. **Mastectomy** is a surgery to remove the entire breast. **Lumpectomy** is a surgery to remove only the breast tumor and a border of healthy tissue around it. This surgery is followed by radiation therapy to kill any cancer cells that may be left in the breast or breast area.

Whether you choose mastectomy or lumpectomy with radiation will not make a difference in how long you live. However, there are other important differences between the surgeries. The decision you make will depend on how you feel about these differences, which include:

- The length of your ***hospital stay***
- The ***discomfort*** you experience after your surgery
- The length of your ***recovery time***
- Whether or not you need ***radiation therapy***
- Whether or not you want to have a second surgery that will re-create the removed breast (this is called ***breast reconstruction***)
- The chance that your cancer may come back in the breast or breast area (this is called ***local recurrence***)
- How your body looks after surgery—i.e. your **appearance**

How you feel about these differences is important, because chances are very good that you will live with the result of your decision for a very long time. To help you make this decision, you will be able to review a website that will provide detailed information about the two surgical treatments. **Please remember that your surgical choice will not affect how long you live.** Because survival after the two treatments is the same for your cancer, your decision should depend on how you feel about the other important differences between the two surgeries. You will be able to spend as much time reviewing the website as you wish; there is no time limit. After you have finished viewing the website, you will be asked to choose the surgery you prefer and to answer several additional questions about your decision.
