# Supplementary figures and images for: The Effect of Patient Narratives on Information Search in a Web-Based Breast Cancer Decision Aid: An Eye-Tracking Study
Source: J Med Internet Res. 2013 Dec 17;15(12):e273. doi: 10.2196/jmir.2784 (PMC3875892; doi:10.2196/jmir.2784)

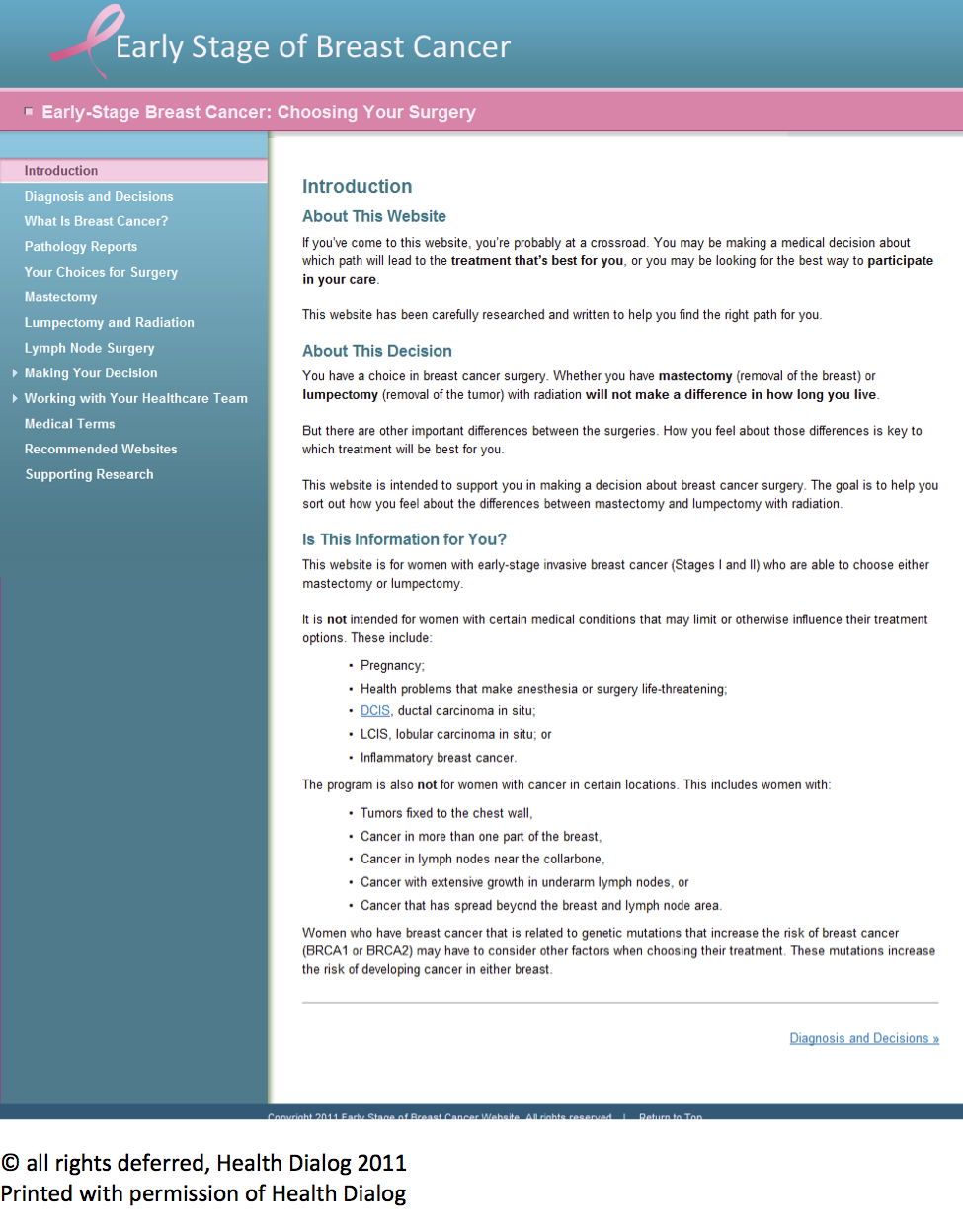

Supplement: Supplementary file 2 [file jmir_v15i12e273_app2.png]

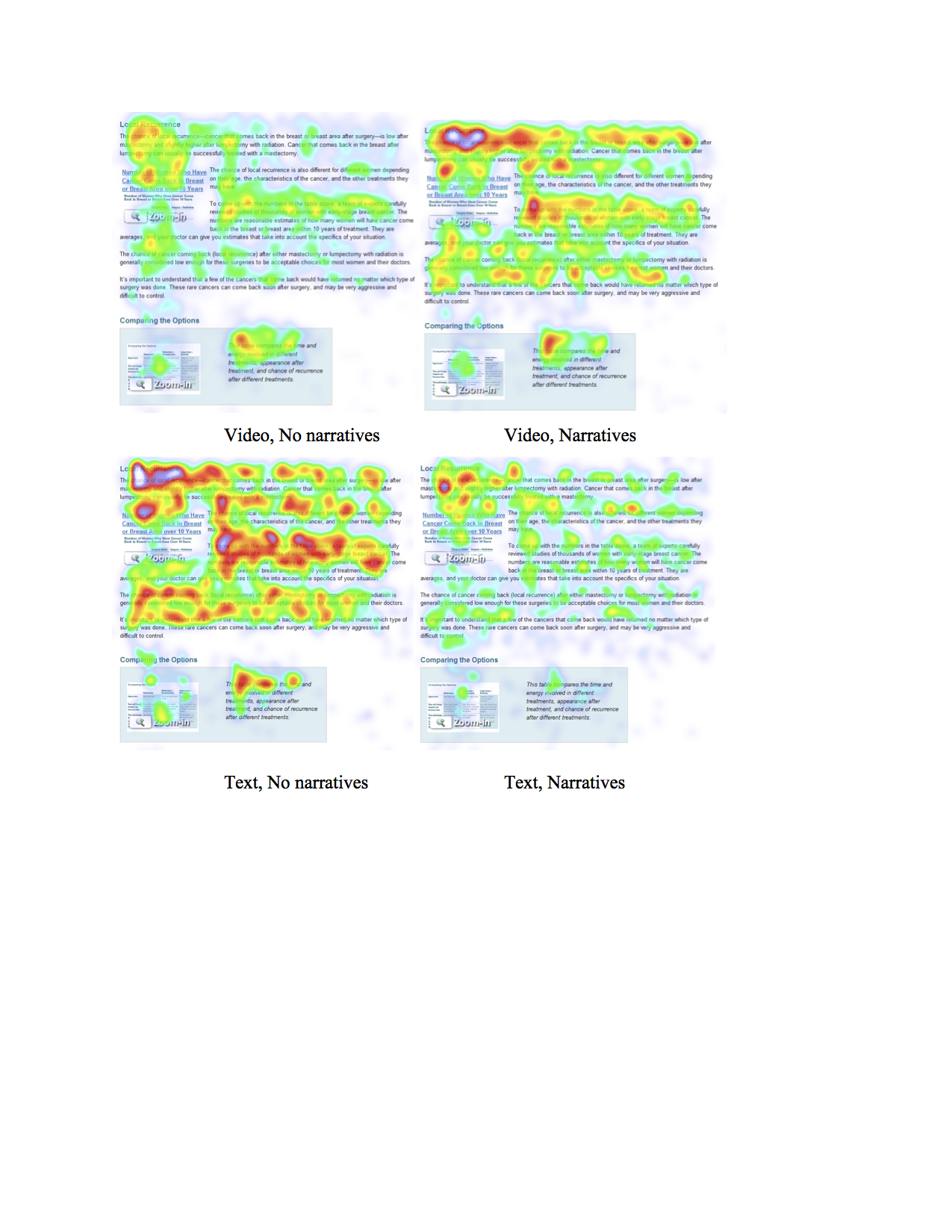

Supplement: Supplementary file 4 [file jmir_v15i12e273_app4.png]

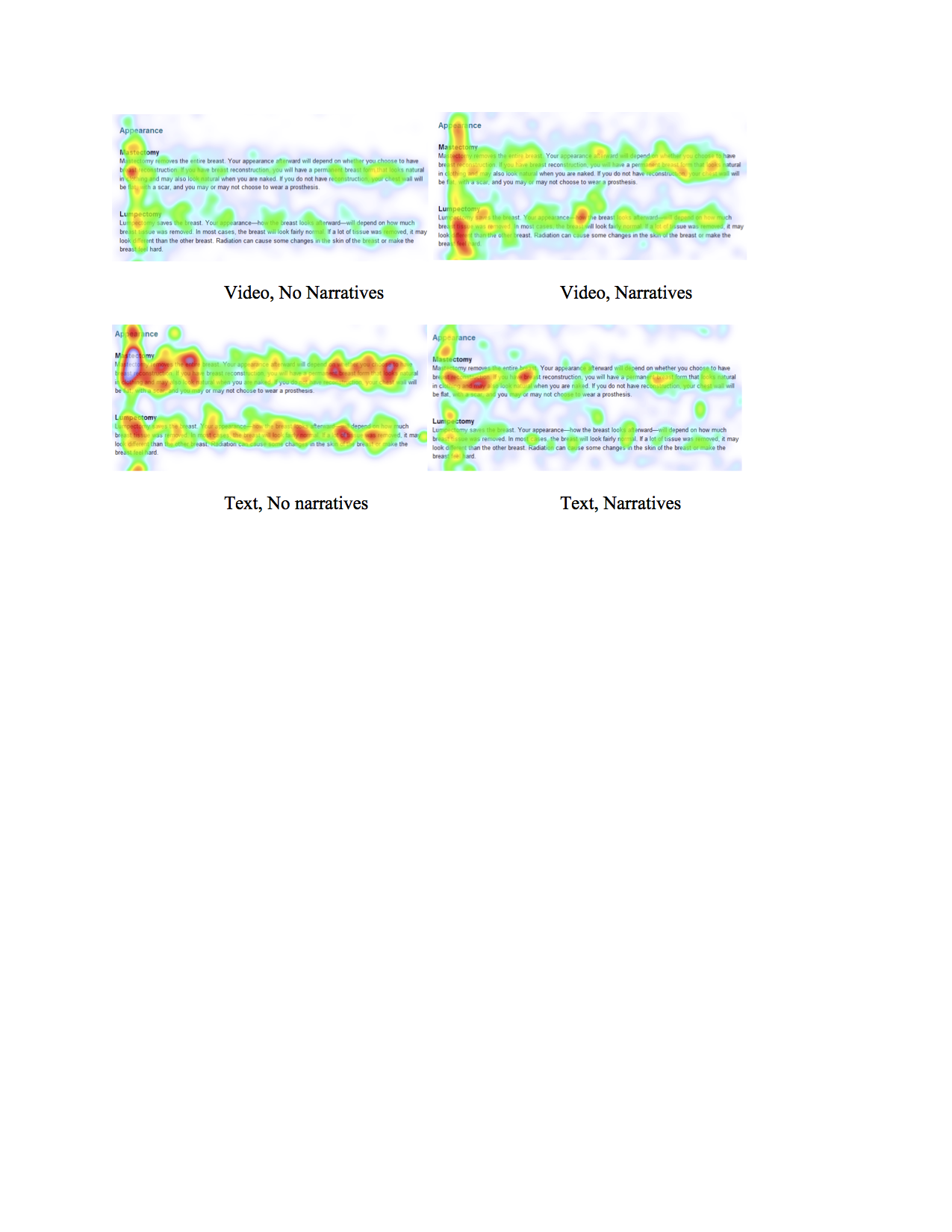

Supplement: Supplementary file 5 [file jmir_v15i12e273_app5.png]
