# Supplementary material for: The Effect of Patient Narratives on Information Search in a Web-Based Breast Cancer Decision Aid: An Eye-Tracking Study
Source: J Med Internet Res. 2013 Dec 17;15(12):e273. doi: 10.2196/jmir.2784 (PMC3875892; doi:10.2196/jmir.2784)
Supplement: Supplementary file 3 [file jmir_v15i12e273_app3.pdf]

Transcript 1: Women and a Doctor Describe Diagnosis

Patient A: He led me into his office, not his examination room and that's when I worried. And he said: "How are you doing?" And I said: "I don't know, you tell me." And he said: "Well I have bad news, you have breast cancer." And I knew and I felt right then that my life was changing and that I would never be the same.

Physician A: There are two major fears that I see women struggling with when they learn they have breast cancer. The first and overweening one is, "Am I going to die of this?" And when they say that they mean "Am I going to die of this soon?" And the second one is, "Am I going to lose my breast?"

Patient B: "I was overwhelmed. I was dealing with already talking about chemo and what type regardless of whether I had any nodes. That was pretty overwhelming. Dealing with having a mastectomy and losing that breast. And "Why?" When there is no "Why." And where do you put this anger that has no target. And death and all of these things."

Patient C: "You know, you're so devastated and you're so mixed up that you don't know what to do. You hear the word "cancer" and you think you're going to be dead within the next few months."

Physician A: "Most women with breast cancer will live out a normal lifespan and have no further difficulty from their breast cancer. It's very hard to react to that in a sensible way emotionally when confronted with breast cancer."

Patient D: "I was like, I'm going to die. I'm going to leave my kids and my grandchildren. I was devastated but once I decided to pull myself together and go look at all the different ways that cancer is cured in terms of the different kinds of cancers and the different kinds of treatments and stuff like that. I felt a whole lot better because then I knew I was going to live."

Transcript 2: Patient A and Patient B Chose Mastectomy

Patient A**:** "While my feeling was the mastectomy wasn't a real big thing. Breasts get in the way. Sometimes they just do. You bump into things, they're in the way. Breasts are good for breast-feeding babies and I'm not going to have any more babies. My boyfriend didn't care whether or not I had them so it was like "Uh, I can do without it". It really wasn't a big thing."

Patient B**:** "I felt the best thing to do, and the most conservative smartest thing, was just to get rid of it. It's bad. Don't take chances. I knew me. I could not live every day wondering, "Oh, is there any left?"

© all rights deferred, Health Dialog 2011
Printed with permission of Health Dialog
